# [SnF(bipy)(H_2_O)]_2_[SnF_6_], a mixed-valent inorganic tin(II)–tin(IV) compound

**DOI:** 10.1107/S2056989024007400

**Published:** 2024-08-06

**Authors:** Natalia Röwekamp-Krugley, Hans Reuter

**Affiliations:** a Chemistry, Osnabrück University, Barbarastr. 7, 49069 Osnabrück, Germany; Harvard University, USA

**Keywords:** mixed-valent, hyper-valency, hydrogen bonding, dimerization, 3*c*–4*e* bonds, crystal structure

## Abstract

In the title compound, bis­[aqua­(2,2′-bi­pyridine)­fluorido­tin(II)] hexa­fluorido­tin(IV), [SnF(C_10_H_8_N_2_)(H_2_O)]_2_[SnF_6_], an ionic mixed-valent tin(II)–tin(IV) compound, the bivalent tin atom is the center atom of the cation and the tetra­valent tin atom is the center atom of the anion. With respect to the first coordination sphere, the cation is monomeric, with the tin(II) atom having a fourfold seesaw coordination with a fluorine atom in an equatorial position, a water mol­ecule in an axial position and the two nitro­gen atoms of the chelating 2,2′-bi­pyridine ligand in the remaining axial and equatorial positions. The bond lengths and angles of this hypervalent first coordination sphere are described by 2*c*–2*e* and 3*c*–4*e* bonds, respectively, all of which are based on the orthogonal 5*p* orbitals of the tin atom. In the second coordination sphere, which is based on an additional, very long tin–fluorine bond that leads to dimerization of the cation, the tin atom is trapezoidal–pyramidally coordinated. The tetra­valent tin atom of the centrosymmetric anion has an octa­hedral coordination. The differences in its tin–fluorine bond lengths are attributed to hydrogen bonding, as the two of the four fluorine atoms are each involved in two hydrogen bonds, linking anions and cations together to form strands.

## Abstract 2

The title compound represents a ionic mixed-valent tin(II)–tin(IV) compound with the bivalent tin atom as central atom of the cation and the tetra­valent tin atom as central atom of the anion. Regarding the first and second coordination sphere, the bivalent tin atom is fourfold, seesaw and fivefold, trapezoid–pyramidal coordinated while the tetra­valent tin atom exhibits an octa­hedral coordination.

## 1. Chemical context

Mixed-valent tin(II)–tin(IV) compounds are most often discovered by chance as a by-product of reactions in which tin(II) compounds are used as starting compounds. Two processes are discussed as potential sources of tetra­valent tin (Gurnani *et al.*, 2013 ▸): the oxidation of divalent tin by atmospheric oxygen and the disproportionation of tin(II) into elemental tin and tin(IV). However, there is no evidence that the reaction proceeds in favor of either process, as the amount of the mixed-valent compound is usually limited to a few crystals. For these reasons, elemental tin is often added to the reaction mixture or an inert gas atmosphere is used.
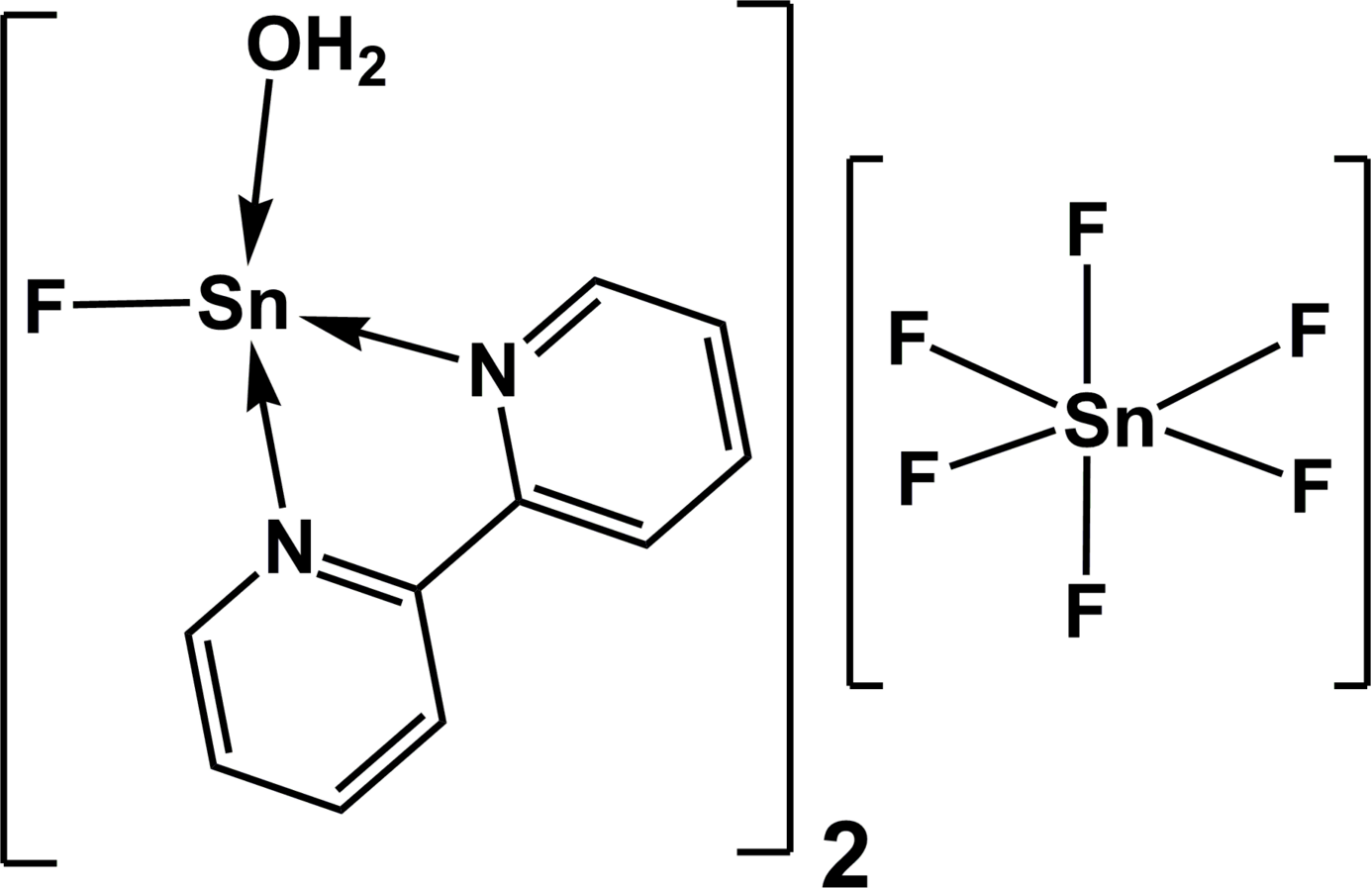


In the case of the title compound, [SnF(H_2_O)(bipy)]_2_[SnF_6_], in a micro-scale experiment in air (and in the absence of elemental tin), we reacted tin difluoride (SnF_2_) with 2,2′-bi­pyridine (bipy) using *N*,*N*-di­methyl­formamide, DMF, as partial solvent and reaction mediator.

## 2. Structural commentary

**Figure 1:**
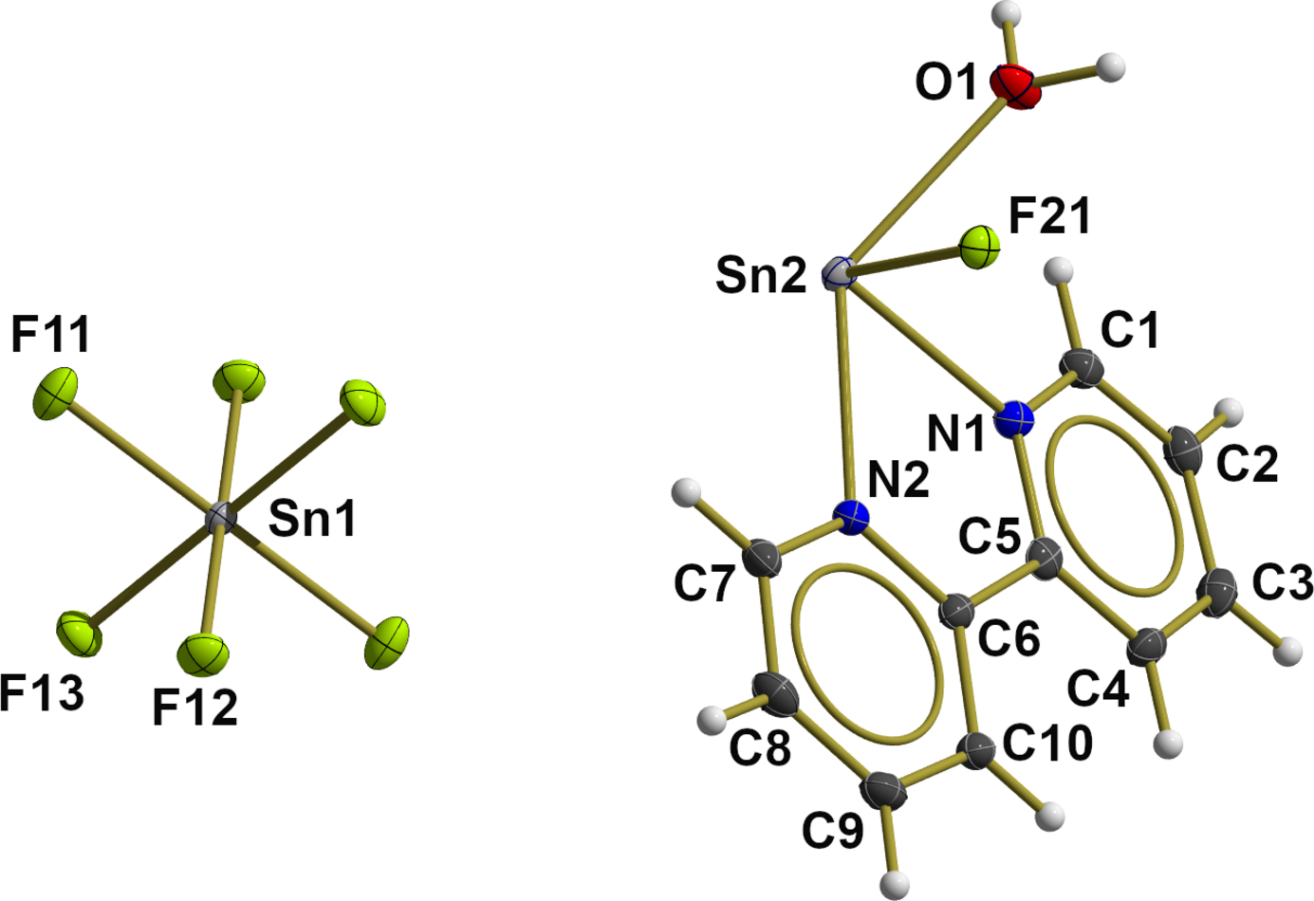
Ball-and-stick model of the ions found in the crystal of the title compound, showing the atom numbering of the asymmetric unit. With the exception of the H atoms, which are shown as spheres of arbitrary radius, all other atoms are drawn with displacement ellipsoids at the 50% probability level.

The title compound crystallizes in the triclinic space group *P*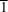 with half a formula unit in the asymmetric unit resulting in a centrosymmetric [Sn^IV^F_6_]^2−^ anion and a [Sn^II^F(H_2_O)(bipy)]^+^ cation in a general position (Fig. 1 ▸).

**Figure 2:**
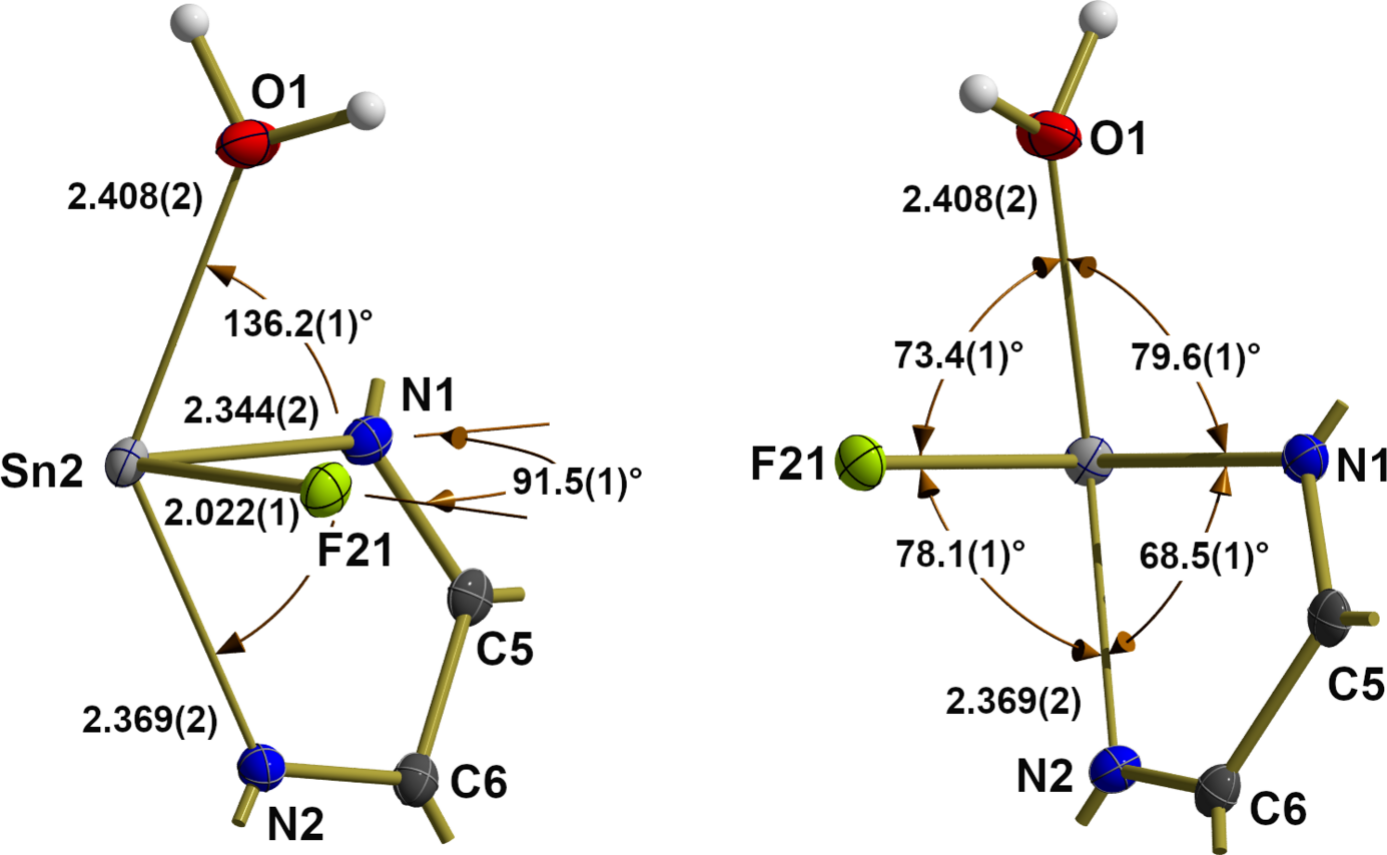
Ball-and-stick model (left: side view; right: front view) of the the first, seesaw coordination sphere of the bivalent tin atom Sn2 of the cation, highlighting selected bond lengths (Å) and angles (°). With the exception of the H atoms, which are shown as spheres of arbitrary radius, all other atoms are drawn with displacement ellipsoids at the 50% probability level. For clarity, only the carbon atoms between the two nitro­gen atoms of the bi­pyridine ligand are shown, the position of all other carbon atoms are indicated as shortened sticks. Axial bonds are drawn as sticks of reduced thickness in order to underline the presence of a 3*c*–4*e* bond.

In the cation, the first coordination sphere of the bivalent tin atom (Fig. 2 ▸) consists of one fluorine atom, the oxygen atom of the water mol­ecule and both nitro­gen atoms of the 2,2′-bi­pyridine ligand. In this seesaw coordination, the fluorine atom [*d*(Sn—F) = 2.022 (1) Å] adopts an equatorial (*eq*) position and the water mol­ecule [*d*(Sn—O = 2.408 (2) Å] an axial (*ax*) one, while the nitro­gen atoms of the chelating 2,2′-bi­pyridine ligand occupy the two other axial and equatorial positions. According with this arrangement, both tin–nitro­gen distances differ [0.025 (1) Å] with the shorter one to N_*eq*_ [2.344 (2) Å] and the longer one to N_*ax*_ [2.369 (2) Å].

Within the equatorial plane, the N_*eq*_—Sn—F bond angle is 91.5 (1)°, whereas the axis is clearly bent [136.2 (1)°]. Among the axial-to-equatorial bond angles, the bond angle between the nitro­gen atoms is very acute [68.5 (1)°] due to the steric restrictions of the ligand. Conspicuously, the bond angle *trans* to this chelating bond angle is also quite acute [73.4 (1)°] while the other two are much more obtuse [78.1 (1)°, 79.6 (1)°].

In the VB concept with its localized 2*c*–2*e* bonds, the bond ratios within the cation are difficult to describe because the tin atom with its non-bonding 5*s* electron pair and the eight electrons of the four donor atoms (F^−^,O,N,N) exceeds the electron octet of a main group element. In contrast, the MO theory according to Pimentel and Rundle (Pimentel, 1951 ▸; Rundle, 1949 ▸) provides a simple and logical explanation for this hyper-valence in the form of a 3*c*–4*e* bond with the exclusive participation of *p* orbitals on all three atoms. In a seesaw-shaped coordination geometry, this 3*c*–4*e* bond is usually found in the axially arranged atoms, while the atoms in the equatorial plane are bonded *via* 2*c*–2*e* bonds through the other *p* orbitals of the tin atom. This bonding concept not only explains the different tin–nitro­gen bond lengths, but also the bond angles around or smaller than 90° as a result of the orthogonality of the tin *p*-orbitals. It also explains the remarkable long [2.408 (2) Å] tin–oxygen distance to the water mol­ecule, for which bond lengths between 2.207 (2) Å and 2.226 (2) Å [mean value: 2.226 (17) Å] are observed (Kleeberg *et al.*, 2022 ▸) in electron-precise tin(II) compounds of the type [Sn(OH_2_)_3_]^2+^ with the tin atom at the apex of a trigonal pyramid and the three oxygen atoms of the water mol­ecules at its base.

**Figure 3:**
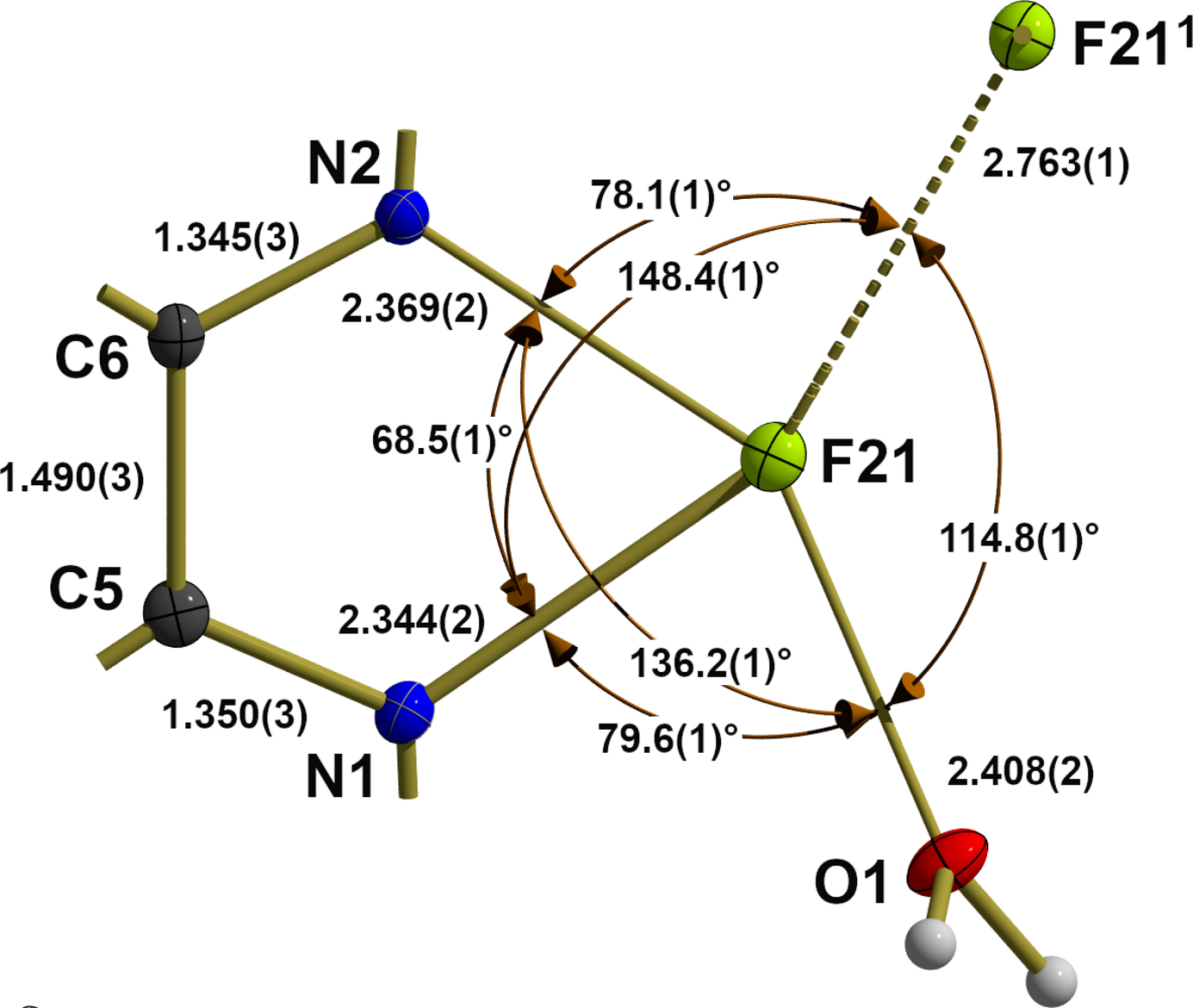
Ball-and-stick model of the second, trapezoid-pyramidal coordination sphere of Sn2, highlighting selected bond lengths (Å) and angles (°), viewed down the fluorine–tin bond. With the exception of the H atoms, which are shown as spheres of arbitrary radius, all other atoms are drawn with displacement ellipsoids at the 50% probability level. For clarity, only the carbon atoms between the two nitro­gen atoms of the bi­pyridine ligand are shown, the position of all other carbon atoms are indicated as shortened sticks. Axial bonds are drawn as sticks of reduced thickness in order to underline the presence of a 3*c*–4*e* bond, as is the additional asymmetric 3*c*–4*e* bond shown with a dashed line. Symmetry code: (i) −*x*, −*y*, 1 − *z*.

**Figure 4:**
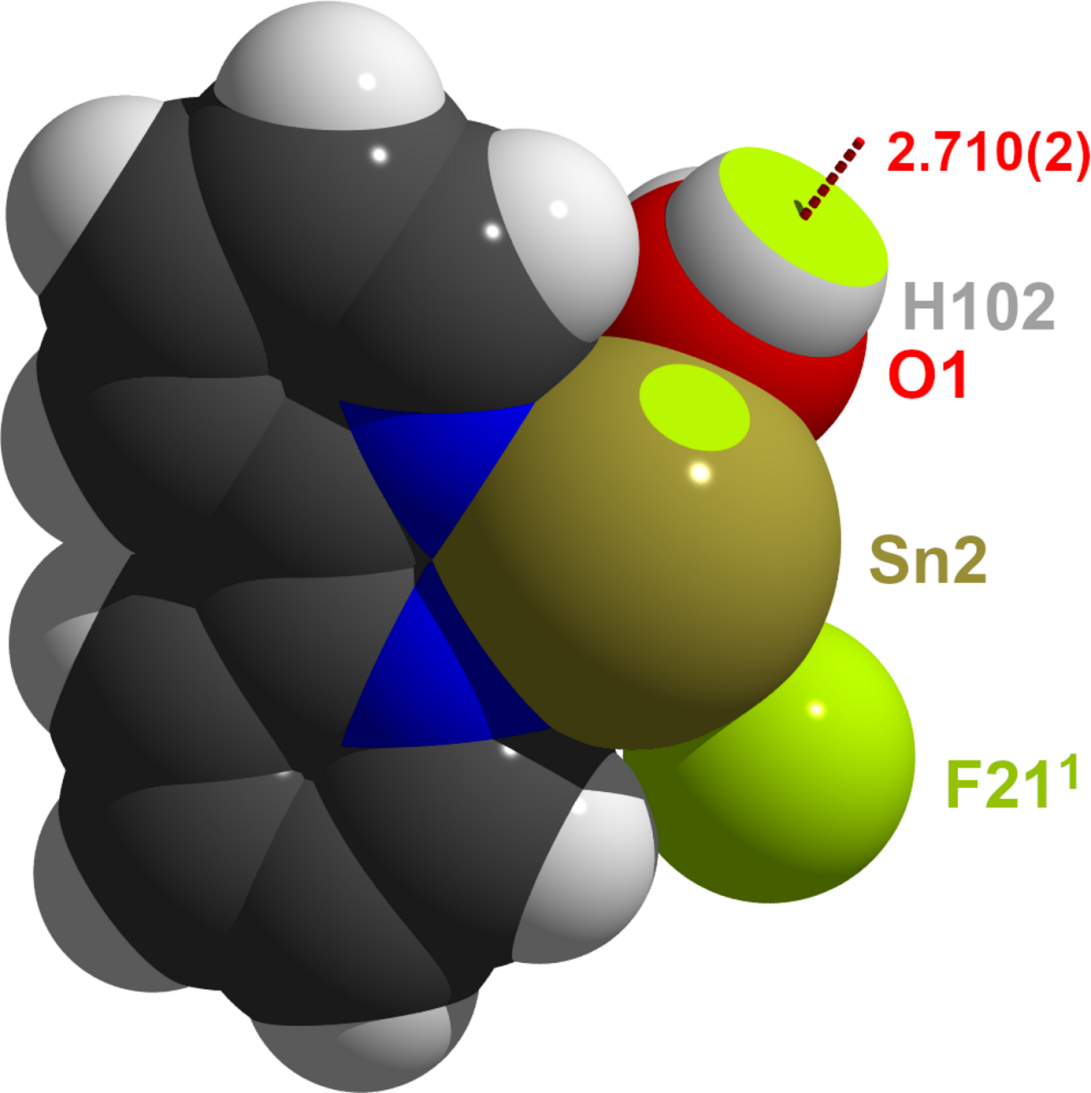
Space-filling model of the [SnF(bipy)(H_2_O)]^+^ cation looking down the *apical* tin–fluorine bond and visualizing the hemi-directed coordination of the bivalent tin atom Sn2. Atoms whose spheres are penetrated by other atoms are visualized as truncated two-colored spheres, the hydrogen bond is indicated by a dashed red line. Atom colors and van der Waals radii (Å) are as follows: F = green/1.47, H = white/1.10, C = gray/1.70, O = red/1.52, N = blue/1.55 and Sn = brass/2.17. Symmetry code: (i) −*x*, −*y*, 1 − *z*.

If only the first coordination sphere is taken as a basis, the cation is monomeric. However, there is a second monomer in its immediate vicinity, whereby both are in contact with each other *via* a long tin–fluorine bridge [*d*(Sn—F) = 2.763 (1) Å] resulting from a center of symmetry (Fig. 3 ▸). This additional, weak bond is in the *trans* position [〈(N—Sn—F) = 148.4 (1)°] to the original, *equatorial* tin–nitro­gen bond and extends the seesaw-shaped, fourfold coordination of the two tin atoms to fivefold, pyramidal coordinations, in which the tin atoms are each below [Δ_least-squares_ = −0.6035 (1) Å] the trapezoidal, strongly uneven [±Δ_max_, _least-squares_ = −0.147 (1)/0.138 (1) Å] base while the shortest bonds [*d*(Sn—F) = 2.022 (1) Å] point in the direction of the *apical* fluorine atoms. This dimerization leads to a bridging angle of 111.8 (1)° at the fluorine atoms and a four-membered, centrosymmetric and therefore exactly planar tin–fluorine ring. Its rhomboidal shape is characterized by acute [68.2 (1)°] angles at the tin atoms and obtuse [111.8 (1)°] ones at the fluorine atoms. Extending the Pimentel–Rundle concept, Musher (1969 ▸) suggests that such additional, very weak bonds result from a *p* orbital of the central atom, that is involved in a classical 2*c*–2*e* bond on the one hand and a 3*c*–4*e* bond on the other. As result, in such ‘asymmetric’ 3*c*–4*e* bonds, one ligand is much more strongly bound to the central atom than the *trans*-ligand. Despite this additional bond, the coordination sphere of the tin atom (Fig. 4 ▸) remains hemi-directed (Shimoni-Livny *et al.*, 1998 ▸).

**Figure 5:**
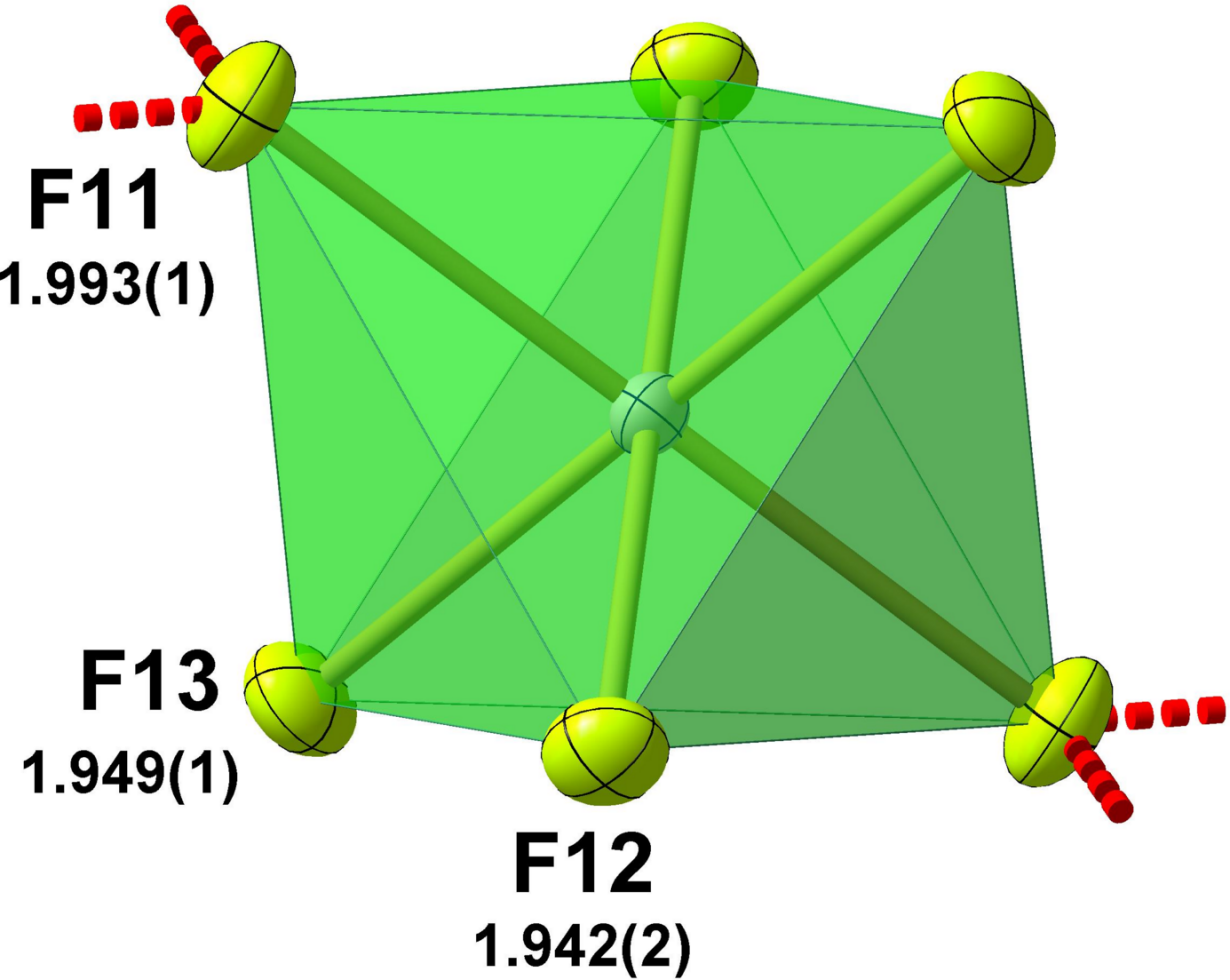
Polyhedron and ball-and-stick model of the octa­hedral, centrosymmetric [SnF_6_]^2−^ anion with bond lengths (Å) and hydrogen bonds indicated by dashed red lines. All atoms are drawn with displacement ellipsoids at the 50% probability level.

The octa­hedral shape of the centrosymmetric [SnF_6_]^2−^ anion is reflected in bond angles around 90° [88.4 (1)–91.6 (1)°] and very similar tin–fluorine bond lengths. While four of the bonds (F12, F13) are almost identical in length [1.949 (1)/1.942 (2) Å], two bonds (F11) are somewhat longer [1.993 (1) Å] because the fluorine atoms undergo hydrogen bonding with the hydrogen atoms of two different water mol­ecules (Fig. 5 ▸).

## 3. Supra­molecular features

**Figure 6:**
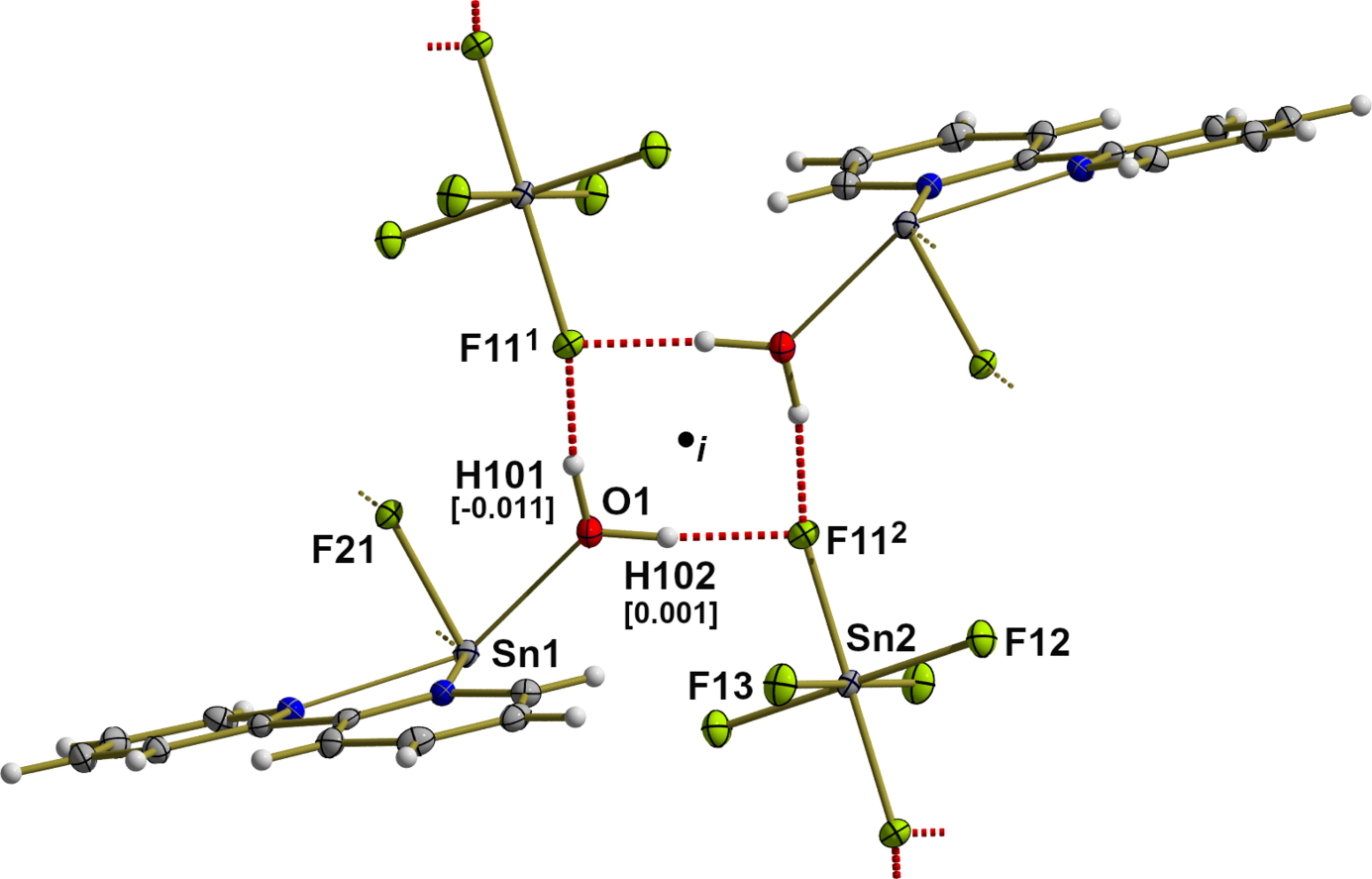
Ball-and-stick model showing in detail the hydrogen-bonding scheme between cation and anion (dashed red lines). With the exception of the H atoms, which are shown as spheres of arbitrary radius, all other atoms are drawn with displacement ellipsoids at the 50% probability level. The out-of-plane deflections (Å) of the hydrogen atoms are given in square brackets, *i* = center of symmetry. Symmetry codes: (i) −*x*, −*y* + 1, −*z* + 1; (ii) *x*, *y*, *z* − 1.

**Figure 7:**
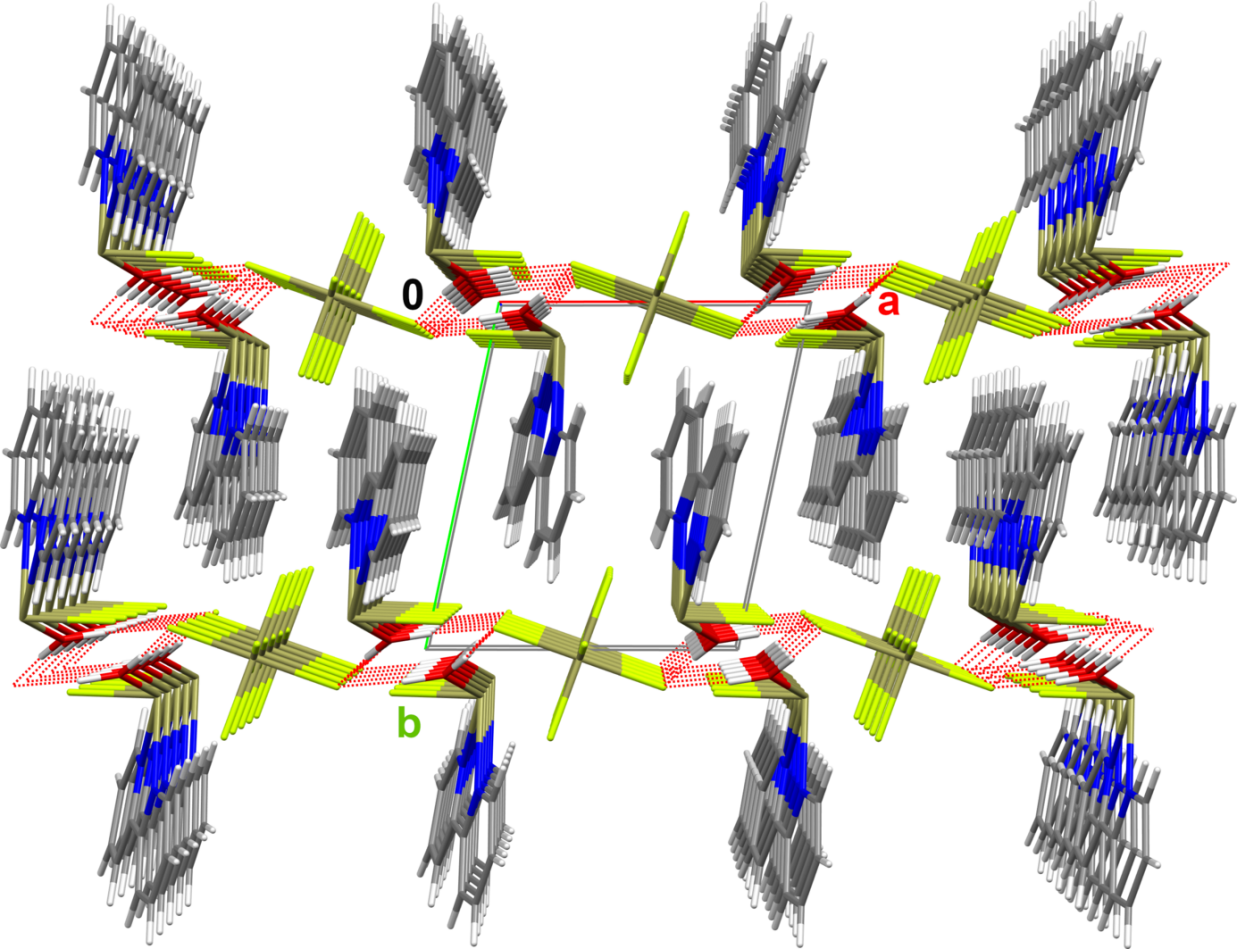
Stick model of the crystal packing looking down the crystallographic *c* axis and showing the strands resulting from the hydrogen bonds (dashed red lines) between anions and cations. Color code as in Fig. 4 ▸.

Similar to the expansion of the coordination spheres of the bivalent tin atoms through further, asymmetric 3*c*–4*e* bonds, the hydrogen bonds between the water mol­ecules of the cation and the fluorine atoms of the anion play a central role in the formation of the crystal structure. Both hydrogen atoms of the water mol­ecule but only one fluorine atom of the anion are involved in these hydrogen bonds whereby a centrosymmetric, almost planar, eight-membered –O—H⋯F ring is formed (Fig. 6 ▸). The geometries of the two crystallographic independent hydrogen bonds are given in Table 1 ▸. The out-of-plane deflections of the H atoms are +0.011 Å for H101 and −0.001 Å for H102. As a result of these hydrogen bonds, the anions and cations are arranged into strands that expand in the *a*-axis direction (Fig. 7 ▸).

## 4. Database survey

The [SnF_6_]^2−^ anion is often a component of salts with organic and inorganic cations. While in combination with inorganic cations the tin–fluorine distances are often strongly influenced by the cation–anion inter­actions [*i.e.* Na_2_SnF_6_: *d*(Sn—F) = 1.958 Å; Benner & Hoppe, 1990 ▸], very similar bond lengths [*d*_mean_(Sn—F) without/with hydrogen bonds: 1.938 (2)/1.956 (2) Å, 24/16 values] to the title compound are found in combination with organic cations (Lermontov *et al.*, 2010 ▸; Bouacida *et al.*, 2005 ▸; Taha *et al.*, 1992 ▸; Cortijo *et al.*, 2017 ▸; Kokunov *et al.*, 2007 ▸; Jung *et al.*, 2023 ▸).

Isolated monomeric or dimeric cations of the type [Sn^II^F(LB^0^)_3_]^+^ or [Sn^II^F(LB^0^)_2_]^+^ with LB^0^ = neutral Lewis base have not yet been described in the literature, but a comparable mixed-valent tin(II)–tin(IV) compound also from SnF_2_ and 2,2′-bi­pyridine was previously described by Gurnani *et al.* (2013 ▸): [SnF(bipy)]_2_[SnF_6_]. In this compound, the first coordination sphere of the bivalent tin atom exhibits a fourfold, seesaw {SnN_2_F_2_} coordination with one additional, axial fluorine atom [*d*(Sn—F) = 2.510 (2) Å] instead of the axial water mol­ecule in the title compound and similar tin–fluorine [*d*(Sn—F)_eq_ = 2.031 (2) Å] and tin–nitro­gen distances [*d*(Sn—N)_eq_ = 2.298 (3) Å, *d*(Sn—N)_ax_ = 2.335 (3) Å]. As in the title compound, dimerization proceeds *via* an asymmetrical 3*c*–4*e* bond [*d*(Sn⋯F_eq_) = 2.738 (3) Å], generating a similar, centrosymmetric four-membered Sn–F ring and trapezoid–pyramidally coordinated tin atoms. Regarding the centrosymmetric [SnF_6_]^2−^ anion of this compound, the Sn—F distances are 1.956 (3) and 1.944 (3) Å for the terminal fluorine atoms and 1.995 (3) Å in case of the μ_2_-fluorine atom that adopts the *axial* position in the first coordination sphere of the bivalent tin atom.

## 5. Synthesis and crystallization

A mixture of approximately 157 mg (1 mmol) of SnF_2_ (Aldrich) and 156 mg (1 mmol) of 2,2′-bi­pyridine (Sigma Aldrich) was placed in a Petri dish and 5 ml of *N*,*N*-di­methyl­formamide (Sigma Aldrich) were added *via* a syringe. No elemental tin was added, nor was oxygen from the air excluded. The following processes were observed from day to day with a light microscope (Stemi 1000, Zeiss, Oberkochen, Germany). After two days small, colorless, block-like crystals of the title compound appeared, which, after two additional days, reached a size that was sufficient for a single crystal X-ray determination.

## 6. Refinement

Crystal data, data collection and structure refinement details are summarized in Table 2 ▸. The positions of all H atoms were clearly identified in difference-Fourier maps. Those of the organic ligand were refined with calculated positions (C—H = 0.93 Å) and a common isotropic displacement parameter. The positions of the H atoms of the water mol­ecule were refined with a fixed O—H distance of 0.96 Å and a bond angle of 104.95° before they were fixed and allowed to ride on the parent O atom with an isotropic displacement parameter.

## Supplementary Material

Crystal structure: contains datablock(s) I. DOI: 10.1107/S2056989024007400/oi2009sup1.cif

Structure factors: contains datablock(s) I. DOI: 10.1107/S2056989024007400/oi2009Isup2.hkl

CCDC reference: 2373684

Additional supporting information:  crystallographic information; 3D view; checkCIF report

## Figures and Tables

**Table 1:** Hydrogen-bond geometry (Å, °)

*D*—H⋯*A*	*D*—H	H⋯*A*	*D*⋯*A*	*D*—H⋯*A*
O1—H101⋯F11^i^	0.96	1.75	2.705 (2)	170
O1—H102⋯F11^ii^	0.96	1.75	2.710 (2)	174

Symmetry codes: (i) 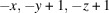; (ii) 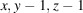.

**Table 2:** Experimental details

Crystal data	
Chemical formula	[SnF(C_10_H_8_N_2_)(H_2_O)]_2_[SnF_6_]
*M*_r_	856.47
Crystal system, space group	Triclinic, *P* 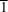
Temperature (K)	100
*a*, *b*, *c* (Å)	7.4579 (4), 8.4601 (5), 9.8864 (6)
α, β, γ (°)	97.073 (2), 98.474 (2), 100.644 (2)
*V* (Å^3^)	599.06 (6)
*Z*	1
Radiation type	Mo *K*α
μ (mm^−1^)	3.19
Crystal size (mm)	0.17 × 0.15 × 0.05

Data collection	
Diffractometer	Bruker APEXII CCD
Absorption correction	Multi-scan (*SADABS*; Krause *et al.*, 2015 ▸
*T*_min_, *T*_max_	0.434, 0.713
No. of measured, independent and observed [*I* > 2σ(*I*)] reflections	28904, 2864, 2611
*R*_int_	0.063
(sin θ/λ)_max_ (Å^−1^)	0.660

Refinement	
*R*[*F*^2^ > 2σ(*F*^2^)], *wR*(*F*^2^), *S*	0.022, 0.053, 1.05
No. of reflections	2864
No. of parameters	172
H-atom treatment	Only H-atom displacement parameters refined
Δρ_max_, Δρ_min_ (e Å^−3^)	0.89, −0.69

Computer programs: *APEX2* and *SAINT* (Bruker, 2009 ▸), *SHELXS97* (Sheldrick, 2008 ▸), *SHELXL2014/7* (Sheldrick, 2015 ▸), *DIAMOND* (Brandenburg, 2006 ▸), *Mercury* (Macrae *et al.*, 2020 ▸) and *publCIF* (Westrip, 2010 ▸).

## supplementary crystallographic information

### Bis[aqua(2,2'-bipyridine)fluoridotin(II)] hexafluoridotin(IV) Crystal data

**Table:**

[SnF(C_10_H_8_N_2_)(H_2_O)]_2_[SnF_6_]	*Z* = 1
*M**_r_* = 856.47	*F*(000) = 406
Triclinic, *P*1	*D*_x_ = 2.374 Mg m^−^^3^
*a* = 7.4579 (4) Å	Mo *K*α radiation, λ = 0.71073 Å
*b* = 8.4601 (5) Å	Cell parameters from 9888 reflections
*c* = 9.8864 (6) Å	θ = 2.5–28.9°
α = 97.073 (2)°	µ = 3.19 mm^−^^1^
β = 98.474 (2)°	*T* = 100 K
γ = 100.644 (2)°	Plate, colourless
*V* = 599.06 (6) Å^3^	0.17 × 0.15 × 0.05 mm

### Bis[aqua(2,2'-bipyridine)fluoridotin(II)] hexafluoridotin(IV) Data collection

**Table:**

Bruker APEXII CCD diffractometer	2611 reflections with *I* > 2σ(*I*)
φ and ω scans	*R*_int_ = 0.063
Absorption correction: multi-scan (SADABS; Krause *et al.*, 2015	θ_max_ = 28.0°, θ_min_ = 3.0°
*T*_min_ = 0.434, *T*_max_ = 0.713	*h* = −9→9
28904 measured reflections	*k* = −10→11
2864 independent reflections	*l* = −13→13

### Bis[aqua(2,2'-bipyridine)fluoridotin(II)] hexafluoridotin(IV) Refinement

**Table:**

Refinement on *F*^2^	Hydrogen site location: mixed
Least-squares matrix: full	Only H-atom displacement parameters refined
*R*[*F*^2^ > 2σ(*F*^2^)] = 0.022	*w* = 1/[σ^2^(*F*_o_^2^) + (0.024*P*)^2^ + 0.4078*P*] where *P* = (*F*_o_^2^ + 2*F*_c_^2^)/3
*wR*(*F*^2^) = 0.053	(Δ/σ)_max_ < 0.001
*S* = 1.05	Δρ_max_ = 0.89 e Å^−^^3^
2864 reflections	Δρ_min_ = −0.69 e Å^−^^3^
172 parameters	Extinction correction: *SHELXL2014/7* (Sheldrick, 2015), Fc^*^=kFc[1+0.001xFc^2^λ^3^/sin(2θ)]^-1/4^
0 restraints	Extinction coefficient: 0.0061 (6)
Primary atom site location: structure-invariant direct methods	

### Bis[aqua(2,2'-bipyridine)fluoridotin(II)] hexafluoridotin(IV) Special details

**Table:**

Geometry. All esds (except the esd in the dihedral angle between two l.s. planes) are estimated using the full covariance matrix. The cell esds are taken into account individually in the estimation of esds in distances, angles and torsion angles; correlations between esds in cell parameters are only used when they are defined by crystal symmetry. An approximate (isotropic) treatment of cell esds is used for estimating esds involving l.s. planes.

### Bis[aqua(2,2'-bipyridine)fluoridotin(II)] hexafluoridotin(IV) Fractional atomic coordinates and isotropic or equivalent isotropic displacement parameters (Å^2^)

**Table:**

	*x*	*y*	*z*	*U*_iso_*/*U*_eq_	
Sn1	0.5000	1.0000	1.0000	0.01059 (8)	
F11	0.22638 (19)	0.91895 (18)	0.96683 (15)	0.0190 (3)	
F12	0.5060 (2)	0.96203 (18)	0.80238 (14)	0.0192 (3)	
F13	0.4618 (2)	1.21909 (17)	0.98795 (15)	0.0198 (3)	
Sn2	0.21949 (2)	0.11033 (2)	0.42958 (2)	0.01159 (8)	
F21	−0.05571 (18)	0.10252 (17)	0.41199 (14)	0.0143 (3)	
N1	0.2719 (3)	0.3727 (2)	0.3702 (2)	0.0124 (4)	
C1	0.3234 (3)	0.3987 (3)	0.2477 (3)	0.0150 (5)	
H1	0.3362	0.3083	0.1854	0.015 (2)*	
C2	0.3578 (3)	0.5513 (3)	0.2103 (2)	0.0161 (6)	
H2	0.3939	0.5660	0.1237	0.015 (2)*	
C3	0.3388 (3)	0.6832 (3)	0.3011 (3)	0.0169 (5)	
H3	0.3597	0.7895	0.2769	0.015 (2)*	
C4	0.2890 (3)	0.6585 (3)	0.4277 (3)	0.0149 (5)	
H4	0.2761	0.7477	0.4914	0.015 (2)*	
C5	0.2581 (3)	0.5018 (3)	0.4601 (2)	0.0125 (5)	
C6	0.2159 (3)	0.4654 (3)	0.5971 (2)	0.0120 (5)	
N2	0.2082 (3)	0.3096 (2)	0.6171 (2)	0.0118 (4)	
C7	0.1811 (3)	0.2676 (3)	0.7394 (2)	0.0152 (5)	
H7	0.1791	0.1582	0.7534	0.015 (2)*	
C8	0.1557 (4)	0.3780 (3)	0.8464 (3)	0.0174 (5)	
H8	0.1343	0.3447	0.9319	0.015 (2)*	
C9	0.1622 (3)	0.5373 (3)	0.8268 (3)	0.0167 (5)	
H9	0.1450	0.6151	0.8991	0.015 (2)*	
C10	0.1940 (3)	0.5838 (3)	0.7003 (2)	0.0135 (5)	
H10	0.2006	0.6934	0.6852	0.015 (2)*	
O1	0.0938 (2)	0.0501 (2)	0.18594 (17)	0.0187 (4)	
H101	−0.0229	0.0670	0.1412	0.073 (10)*	
H102	0.1481	0.0034	0.1126	0.073 (10)*	

### Bis[aqua(2,2'-bipyridine)fluoridotin(II)] hexafluoridotin(IV) Atomic displacement parameters (Å^2^)

**Table:**

	*U*^11^	*U*^22^	*U*^33^	*U*^12^	*U*^13^	*U*^23^
Sn1	0.00996 (13)	0.01193 (14)	0.01123 (13)	0.00387 (9)	0.00360 (9)	0.00239 (9)
F11	0.0116 (7)	0.0237 (8)	0.0199 (8)	0.0027 (6)	0.0030 (6)	−0.0024 (6)
F12	0.0216 (8)	0.0231 (8)	0.0133 (7)	0.0042 (6)	0.0053 (6)	0.0027 (6)
F13	0.0255 (8)	0.0155 (8)	0.0224 (8)	0.0096 (6)	0.0069 (6)	0.0062 (6)
Sn2	0.01071 (11)	0.01044 (11)	0.01397 (12)	0.00390 (7)	0.00208 (7)	0.00069 (7)
F21	0.0110 (7)	0.0161 (7)	0.0165 (7)	0.0044 (5)	0.0035 (5)	0.0015 (6)
N1	0.0104 (10)	0.0133 (10)	0.0134 (10)	0.0023 (8)	0.0027 (8)	0.0004 (8)
C1	0.0130 (12)	0.0165 (13)	0.0145 (13)	0.0023 (10)	0.0017 (10)	0.0009 (10)
C2	0.0120 (13)	0.0223 (15)	0.0150 (14)	0.0043 (11)	0.0022 (11)	0.0054 (12)
C3	0.0146 (12)	0.0152 (13)	0.0214 (14)	0.0020 (10)	0.0025 (10)	0.0071 (11)
C4	0.0138 (12)	0.0124 (12)	0.0186 (13)	0.0050 (9)	0.0019 (10)	0.0003 (10)
C5	0.0066 (11)	0.0152 (12)	0.0161 (12)	0.0031 (9)	0.0020 (9)	0.0023 (10)
C6	0.0091 (11)	0.0115 (12)	0.0145 (12)	0.0020 (9)	0.0004 (9)	0.0009 (10)
N2	0.0128 (10)	0.0107 (10)	0.0111 (10)	0.0024 (8)	0.0003 (8)	0.0010 (8)
C7	0.0156 (12)	0.0148 (12)	0.0132 (12)	0.0008 (10)	−0.0010 (10)	0.0024 (10)
C8	0.0168 (13)	0.0225 (14)	0.0118 (12)	0.0012 (10)	0.0026 (10)	0.0027 (11)
C9	0.0156 (12)	0.0190 (13)	0.0141 (13)	0.0044 (10)	0.0014 (10)	−0.0027 (10)
C10	0.0136 (12)	0.0121 (12)	0.0142 (12)	0.0032 (9)	0.0009 (9)	0.0000 (10)
O1	0.0162 (9)	0.0269 (10)	0.0132 (9)	0.0068 (8)	0.0037 (7)	−0.0016 (8)

### Bis[aqua(2,2'-bipyridine)fluoridotin(II)] hexafluoridotin(IV) Geometric parameters (Å, º)

**Table:**

Sn1—F13	1.9418 (14)	C3—H3	0.9500
Sn1—F13^i^	1.9418 (14)	C4—C5	1.389 (3)
Sn1—F12^i^	1.9491 (14)	C4—H4	0.9500
Sn1—F12	1.9491 (14)	C5—C6	1.490 (3)
Sn1—F11	1.9931 (14)	C6—N2	1.349 (3)
Sn1—F11^i^	1.9931 (14)	C6—C10	1.389 (3)
Sn2—F21	2.0221 (13)	N2—C7	1.335 (3)
Sn2—N1	2.344 (2)	C7—C8	1.381 (4)
Sn2—N2	2.369 (2)	C7—H7	0.9500
Sn2—O1	2.4076 (17)	C8—C9	1.378 (4)
N1—C5	1.350 (3)	C8—H8	0.9500
N1—C1	1.354 (3)	C9—C10	1.396 (3)
C1—C2	1.375 (3)	C9—H9	0.9500
C1—H1	0.9500	C10—H10	0.9500
C2—C3	1.386 (3)	O1—H101	0.9600
C2—H2	0.9500	O1—H102	0.9600
C3—C4	1.385 (4)		
			
F13—Sn1—F13^i^	180.0	C4—C3—C2	119.3 (2)
F13—Sn1—F12^i^	89.28 (6)	C4—C3—H3	120.3
F13^i^—Sn1—F12^i^	90.72 (6)	C2—C3—H3	120.3
F13—Sn1—F12	90.72 (6)	C3—C4—C5	119.1 (2)
F13^i^—Sn1—F12	89.28 (6)	C3—C4—H4	120.4
F12^i^—Sn1—F12	180.00 (9)	C5—C4—H4	120.4
F13—Sn1—F11	89.59 (6)	N1—C5—C4	121.6 (2)
F13^i^—Sn1—F11	90.41 (6)	N1—C5—C6	115.7 (2)
F12^i^—Sn1—F11	88.39 (6)	C4—C5—C6	122.7 (2)
F12—Sn1—F11	91.61 (6)	N2—C6—C10	121.7 (2)
F13—Sn1—F11^i^	90.41 (6)	N2—C6—C5	115.3 (2)
F13^i^—Sn1—F11^i^	89.59 (6)	C10—C6—C5	122.9 (2)
F12^i^—Sn1—F11^i^	91.62 (6)	C7—N2—C6	119.5 (2)
F12—Sn1—F11^i^	88.39 (6)	C7—N2—Sn2	120.78 (16)
F11—Sn1—F11^i^	180.00 (3)	C6—N2—Sn2	119.55 (15)
F21—Sn2—N1	91.52 (6)	N2—C7—C8	122.1 (2)
F21—Sn2—N2	78.11 (6)	N2—C7—H7	118.9
N1—Sn2—N2	68.50 (7)	C8—C7—H7	118.9
F21—Sn2—O1	73.42 (6)	C9—C8—C7	118.8 (2)
N1—Sn2—O1	79.62 (6)	C9—C8—H8	120.6
N2—Sn2—O1	136.22 (6)	C7—C8—H8	120.6
C5—N1—C1	118.7 (2)	C8—C9—C10	119.7 (2)
C5—N1—Sn2	120.40 (15)	C8—C9—H9	120.2
C1—N1—Sn2	120.81 (16)	C10—C9—H9	120.2
N1—C1—C2	122.4 (2)	C6—C10—C9	118.1 (2)
N1—C1—H1	118.8	C6—C10—H10	121.0
C2—C1—H1	118.8	C9—C10—H10	121.0
C1—C2—C3	118.8 (2)	Sn2—O1—H101	127.2
C1—C2—H2	120.6	Sn2—O1—H102	127.9
C3—C2—H2	120.6	H101—O1—H102	105.0

Symmetry code: (i) −*x*+1, −*y*+2, −*z*+2.

### Bis[aqua(2,2'-bipyridine)fluoridotin(II)] hexafluoridotin(IV) Hydrogen-bond geometry (Å, º)

**Table:**

*D*—H···*A*	*D*—H	H···*A*	*D*···*A*	*D*—H···*A*
O1—H101···F11^ii^	0.96	1.75	2.705 (2)	170
O1—H102···F11^iii^	0.96	1.75	2.710 (2)	174

Symmetry codes: (ii) −*x*, −*y*+1, −*z*+1; (iii) *x*, *y*−1, *z*−1.
